# Ensemble machine learning and forecasting can achieve 99% uptime for rural handpumps

**DOI:** 10.1371/journal.pone.0188808

**Published:** 2017-11-28

**Authors:** Daniel L. Wilson, Jeremy R. Coyle, Evan A. Thomas

**Affiliations:** 1 Civil & Environmental Engineering, University of California, Berkeley, California, United States of America; 2 Biostatistics, University of California, Berkeley, California, United States of America; 3 Mechanical Engineering, Portland State University, Portland, Oregon, United States of America; 4 SweetSense Inc., Portland, Oregon, United States of America; TNO, NETHERLANDS

## Abstract

Broken water pumps continue to impede efforts to deliver clean and economically-viable water to the global poor. The literature has demonstrated that customers’ health benefits and willingness to pay for clean water are best realized when clean water infrastructure performs extremely well (>99% uptime). In this paper, we used sensor data from 42 Afridev-brand handpumps observed for 14 months in western Kenya to demonstrate how sensors and supervised ensemble machine learning could be used to increase total fleet uptime from a best-practices baseline of about 70% to >99%. We accomplish this increase in uptime by forecasting pump failures and identifying existing failures very quickly. Comparing the costs of operating the pump per functional year over a lifetime of 10 years, we estimate that implementing this algorithm would save 7% on the levelized cost of water relative to a sensor-less scheduled maintenance program. Combined with a rigorous system for dispatching maintenance personnel, implementing this algorithm in a real-world program could significantly improve health outcomes and customers’ willingness to pay for water services.

## Introduction

Reliable and sustained water service delivery for the global poor remains a significant challenge [1]. In emerging economies, today’s most common approach for delivering water services focuses on deploying, maintaining, and monitoring aid projects for a short period of time. Maintenance and impact evaluation is nominally performed by implementers (non-profits, private companies, and governments alike), but often these infrastructure and aid projects fail after short-term subsidies and supervision lapses. For example, in rural sub-Saharan Africa, a large proportion of hand-operated pumps (handpumps) are broken; the Rural Water Supply Network estimates that 10-67% of improved water sources are non-functional at any given time [2], and much of this infrastructure will never be repaired.

Even when pumps work, only half of functioning handpumps adequately meet the implicit intent of the World Health Organization’s Millennium Development Goal 7 (MDG 7): “ensure environmental sustainability, targeting safe drinking water” [3]. The handpumps fail to meet the MDG 7 because they often deliver poor-quality groundwater or drive users to consume surface water during outages resulting from pump damage or seasonal fluctuations in water table levels [4]. Finally, imprecise methods used by non-profits, private companies, and well drillers to evaluate handpumps before and after implementation adds to the reduction of true water access improvements figures in developing countries [5]. Hence, there is increasing evidence that much of the water, sanitation and energy services provided in developing countries have failed to keep the promise of delivering clean water over decadal time scales.

Under today’s industry-standard service delivery model, a water pump implementation agency will repair pumps when a community contact calls to report a pump failure (if the implementation agency is still the active steward of the pump). However, this model breaks down if community members do not initiate communication with the implementation agency, or if the implementation agency does not respond to community requests because of a lack of accountability. By contrast, in previous work we used sensor data and a public dashboard for pump failure identification and accountability [6]. The model to identify pump failures was very simple: uncharacteristically long gaps in pump use were used as a pump failure heuristic, and service was dispatched accordingly. This pump maintenance model was termed the “ambulance model” and resulted in an average of 91% of the pump fleet functioning at any given time compared to just 56% of pumps functioning under the industry-standard service model [6].

Achieving even higher fleet uptime (>99%) is seen as a critical step towards healthy and financially-sustainable water delivery. Intermittent access to clean water is known to substantially increase health risks; drinking clean water 9 out of 10 weeks is not enough to prevent diarrheal and other disease. However, substantial gains in health outcomes can be realized if infrastructure delivers clean water >99% of the time. A recently-published model compared the health impacts (in terms of averted disability-adjusted life-years, ADALYs) between consumption of untreated water and consumption of water free of microbial contamination. The model found that “high adherence” to consuming clean drinking water yielded dramatic improvements in health outcomes relative to “moderate” adherence [7]. Additionally, many stakeholders believe that high reliability is key to unlocking consumers’ willingness to pay for improved water service delivery; working pumps may illicit a virtuous cycle where reliable water delivery unlocks payments which in turn are used to maintain pumps [8].

Although sustained high-quality water delivery requires the entire system of water delivery to function effectively (i.e. a “systems thinking” approach to water delivery) [9], data from sensors has demonstrated substantial increases in fleet performance. In our prior work, we implemented the “ambulance maintenance model” where sensors enabled as-needed pump repairs. This maintenance model led to about 9% of pumps being broken on average, and the primary driver of downtime was the lag time between when a pump failed and when it was repaired. This lag time is a result of the combination of the time it took for the failure heuristic to identify a failure and the delay in dispatching a repair person; it typically took 21 days between when a pump failed and when a successful repair in the field was performed.

To boost uptime from 91% to >99%, we need a new preventative maintenance framework that services pumps as soon as, or ideally before, they fail. Often, preventative maintenance is operationalized by servicing equipment on a fixed schedule [10, 11]. However, a campaign of routine scheduled maintenance and service in rural Africa could be expensive and cumbersome; sending maintenance workers to the field to service working pumps is a drain on implementation agencies’ limited resources. Instead, agencies would prefer to identify pumps at high risk for failure and service them as-needed and “just-in-time” before they break.

This condition-based maintenance, or the dispatch of maintenance resources in response to a measured or predicted fault, has several advantages over time-based (scheduled) maintenance. The most important advantage of condition-based maintenance is the ability to allocate limited maintenance resources where they are needed instead of spreading maintenance resources evenly, including where they may not be needed [12]. A key factor in condition-based maintenance is the estimation of remaining useful life (RUL) which is the amount of time left before a particular device fails or requires maintenance. Techniques for estimation of RUL fall largely into two groups: physical models (based on a predefined relationship between physical properties of a device and failure), and data-driven statistical (probabilistic) or machine learning (predictive) models [13]. Such techniques have been applied in a wide variety of industries [14] including aerospace [15], computing [13], and electrical distribution [16].

In this study, we explore the possibility of a preventative maintenance of handpumps enabled by machine learning. We train a model based on human-verified and sensor-observed pump failures, and we analyze the ability of the learner to forecast failures and to identify failures quickly after they happen. We use the results of this learner to assess the impacts of a hypothetical implementation of the scheme on a real-world pump fleet in western Kenya. We analyze the hypothetical performance of the fleet as well as estimated impacts on costs to the implementation agency and the lifetime cost of pump operation.

## Design and methods

### Study population

For this study, we analyzed a group of 42 Afridev-brand handpumps in western Kenya. The pumps are maintained by The Water Project, “a 501(c)(3) non-profit organization unlocking human potential by providing reliable water projects to communities in sub-Saharan Africa who suffer needlessly from a lack of access to clean water and proper sanitation.” All pumps were monitored by a SweetSense-brand cellular data-enabled handpump sensor. Data on 3-axis acceleration (measuring standpipe vibrations inducted by pumping activity) and pump basin gauge pressure (a proxy for flowrate out of the nozzle attached to the water basin) were logged at 10-second intervals while there was activity at the pump. This strategy reduced data volumes by not sending 10-secondly data for times where the pump is not in use. For this study, we used sensor data from mid-January 2016 through March 2017, for a total of 8962 sensor-observed pump days (not all pumps were monitored by sensors for the full study).

### Super learner

We employed a supervised ensemble machine learning tool, Super Learner [17], for predicting pump failures. Super Learner employs an ensemble of robust machine learning classification techniques, using cross-validation methods to tune model parameters. We employed a number of candidate machine learning algorithms (“learners”), including simple regression models, Support Vector Machines, [18] Multivariate Adaptive Regression Splines, [19] and Random Forests [20]. We then combined estimates from this ensemble of learning methods using cross-validation. Cross validation masks random sections of the training set (pump-wise in our case) and tests the performance of a learner trained on the remaining data against the masked data [21]. In this way, the full dataset is both the training and the testing set, but cut into multiple random subsets to be used as training and testing. This technique is the best practice to avoid over-fitting in supervised machine learning.

This strategy allowed us to find optimal convex combination (weighted average) of an ensemble of candidate prediction algorithms (i.e. model stacking/Super Learning) [17, 22]. Our classifier is probabilistic, but the eventual output of the machine learning tool is a thresholded binary outcome: to dispatch a service person or not. The goal of this technique was to create a data-adaptive system capable of predicting failure well enough in advance to allow preventive maintenance, repair, or replacement.

### Training

The Water Project keeps detailed records of pumps service visits, and these records served as a training set for the Super Learner. Ground-truth data about when and why pumps failed was used to classify a training set spanning the entire duration of the sensor-observed dataset. The Water Project’s records indicate when a pump site was visited, what was wrong (if anything), and when the pump was repaired (if applicable). However, the Water Project’s in-field records do not contain precise start dates for pump failures. This lack of information is expected because field workers can only confirm that pumps are broken and record when pumps are repaired; it is difficult for field staff to define precisely when a broken pump’s problems began. Therefore, manual inspection of sensor data was used to estimate the start time for field-verified pump failures.

In addition to field-verified pump failures, several events of persistent pump disuse occurred that were not represented in failure records by The Water Project. These events could have represented a short-term pump failure that did not initiate a service call (such as silt buildup or low water table), or could have been due to cultural or environmental factors (disuse due to holidays or heavy rain, for example). Regardless, these events were difficult to distinguish from sudden pump failures. Because of the nature of these events, we believed that they still warranted service call or site visit and thus needed prediction, so we also manually classified them as “failures.” In total, 24 pump failure events were identified. These failures ranged from 1 to 42 days in length, with a mean and standard deviation of 12.0 and 9.8 days, respectively. This amounts to 288 pump failure days observed over the 8962 pump-day observation period.

The 24 observed failure events were used to train the learner. Although the handpump data was collected at 10-second intervals, the training set for the learner was defined in terms of calendar days. The positive class of the “current pump failure” training set was any calendar day for which a failure event was ongoing. In some studies, times series data (such as pump vibration and pressure) are labeled using the positive or negative class, but our data collection strategy did not send data to our servers when the pump was not in use. For this reason, “broken days” made more sense as a positive training class than “broken data points.” We chose to train using broken days versus broken hours/minutes/seconds because of (1) an *a priori* assumption that the learner could not generate accurate predictions on time resolutions of less than a day, and (2) the reality that implementers can not take action on pump failure information at time scales less than a day.

To train the learner for forecasting failures, we programmatically defined seven different positive (“will fail”) training classes ranging from 1-7 calendar days before the start date of any pump failure (e.g. “pump will fail in 3 days”). Each of these 7 training classes trained their own separate Super Learner, then the predictions of these 7 learners were pooled and used as covariates in a final “forecast failure” learner.

### Feature definition

Using the raw pressure and acceleration time series data, we defined the concept of a “pumping event.” Vibration and head pressure in the water basin were measured by a SweetSense unit as proxies for handle motion and water flow, respectively. The start of an event is defined from when the pump’s handle begins to move and ends when the handle stops moving for 3 or more minutes. For each pumping event, we calculated summary metrics. Then, we rolled up event-wise metrics into day-wise features that, based on domain knowledge, we believed were predictive of imminent pump failure. Because all features are defined calendar-day-wise, all of the learners’ predictions are also calendar-day-wise.

**Fig 1:**
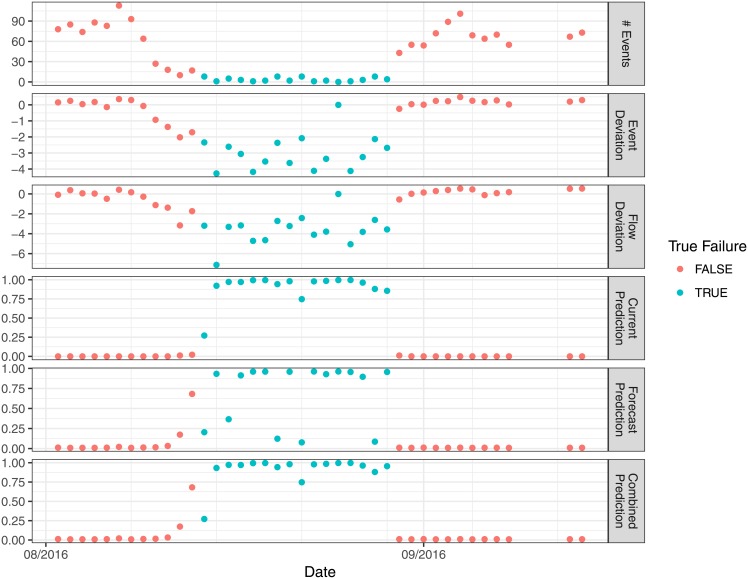
Features and model outputs plotted as time series. All data is colored by field-verified failure status. Three features are plotted: number of events is the number of pumping events counted in a given day, while event deviation and flow deviation are normalized log-scale deviation of an event’s duration and flow from its expected duration (correcting for day of week). Following the features are the outputs of the current failure prediction, the forecast failure prediction, and the combined prediction (max of current and forecast). The y-axis of these metrics can be thought of as the probability that the pump has failed (current prediction), will fail (future forecast prediction), or has/will fail (combined prediction). For a threshold probability of 0.5, a future failure would be forecast the day before the true failure occurred for both the forecast classifier and the combined classifier.

Feature extraction using domain knowledge is a common technique in machine learning that reduces complex raw data into a smaller set of relevant variables that have a more simple and consistent relationship with the outcome of interest, in our case device failure [23]. We identified nine features that were of significant predictive value. These features broke down into four categories: features based on the number of pumping events per day, the pump’s flow rate, the duration of pumping events, and the ratio of a pump’s flow rate to amount of handle motion (i.e. volume of water per human effort). For each category, we defined features based not only on the per-event metric, but also several measures of deviation between the per-event metric and the expected value (based on recent historical data). The three most important are plotted in Fig 1. We were surprised to find that, often times, the features that represented the absolute value of a physical property (e.g. flow) were often weighted less heavily than the same property’s deviation from a recent historical trends. Although its not possible to know how certain features would have been weighted with a larger data set, our study points to the potential value of features that rate historically-relative properties of a pump rather than just the properties themselves. It is interesting to note that some of these features capture physical properties of our pumps, and therefore our model is not a purely statistical model, but partially a physical model.

### Learner customization

The learner operates in two-stages. The first stage of the learner makes predictions day-wise; only data from that particular day is used to estimate whether the pump has or will fail. In the second stage, the learner accounts for long periods of time. For example, in its second stage the learner may discount the probability of a single failed day islanded in a long stretch of working days. The final predictors of current and forecast failure used for further analysis are given by the output of the second stage. The model was unable to forecast failure with any better capability than a random guess at forecast horizons beyond 7 days.

The current and forecast learners do not directly predict the binary outcome of “not failed vs. failed” or “won’t fail vs. will fail.” Instead, they provide a probability of failure for each day. Any given pump-day will fall on a probability scale from 0-1 both in terms of the probability that the pump is currently failed (current failure prediction), or will fail within 7 days (forecast failure prediction). This probability that a pump has/will fail on any given day is illustrated in the bottom three panels of Fig 1.

To operationalize the learner and turn its output into a binary “not failed vs. failed” or “won’t fail vs. will fail” outcome, the implementer sets a threshold between 0 and 1 on the output of the second stage prediction; above the threshold, the implementer assumes that the pump has/will fail, and below the threshold the implementer assumes the pump has/will not fail. The closer to 1 this threshold is set, the fewer false positives (believing the pump is broken when it is not) will be classified, but this will be balanced with an increased number of false negatives (thinking the pump is working when it has actually failed).

### Implementation assumptions

Several assumptions had to be made about how an implementing agency would incorporate this learner and how preventative maintenance would affect pump performance. First, we analyzed a range of dispatch delays from 1 day to 21 days. We define the dispatch delay as the amount of time between when the model identifies a pump failure and when a “dispatch” takes place. In this context, a dispatch could be any action from a phone call to sending a maintenance worker to the site.

Next, we needed to consider what would happen in the case of a dispatch resulting from a forecasted failure. If a service person called or visited the site with the forecasted failure, but the pump seemed to be working well, the service person would assume (rightly) that the forecasting model had generated a false positive, and would leave without performing maintenance. Conversely, a service person who arrived on site to find a deteriorating (but not quite broken) pump would assume the model had correctly forecasted a failure and perform preventative maintenance. For this study, we assume that a pump that is within one week of a true failure will show significant enough deterioration that the service person will perform preventative maintenance, thus preventing the future failure. In other words, we assume that a preventative maintenance service visit only prevents failures that would have happened within one week of the service visit. Thus, this assumption is conservative.

Finally, we assumed that in a real-world implementation, there would be a re-dispatch interval. For example, if our model identified a false positive failure on a Monday and a service person arrived on site to find a pump working well, it would not be reasonable to expect that service person to re-dispatch on Tuesday if the model still predicted a failure. For this study, we assume a one week re-dispatch interval before a service person would re-dispatch to the same site.

## Results

**Fig 2:**
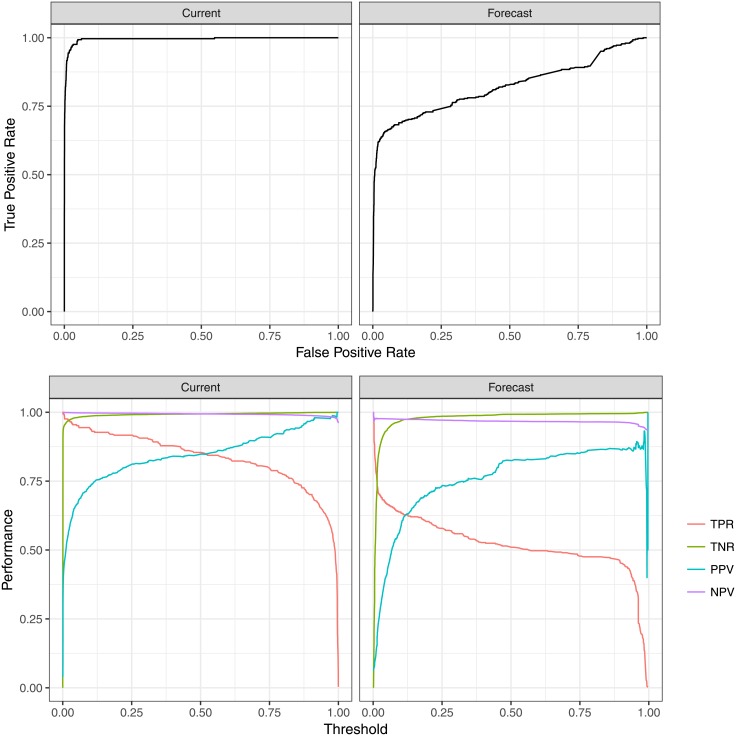
Performance of the ensemble learner. Top: the Receiver Operating Characteristic (ROC) curve for both current and forecast failure prediction. The ROC represents the range of possible trade-offs between the classifiers’ true positive (truly failed pump classified as a failure) and false positive (truly functional pump classified as a failure) rates when choosing a threshold to operationalize the classifier. Bottom: the true positive rate (TPR), true negative rate (TNR), positive predictive value (PPV), and negative predictive value (NPV, not to be confused with net present value) are plotted as a function of the learner’s probability threshold. This bottom panel illustrates the relationship between learner performance and the implementer-defined probability threshold to decide of a pump is broken (current) or will break (forecast) on any given day.

The top panel of Fig 2 illustrates the learners’ receiver operating characteristic which show the trade-off between true positive rate (TPR) and false positive rate (FPR). In this context, a “true positive” occurs when the classifier correctly (true) identifies a pump failure (condition positive), and a “false positive” occurs when the classifier incorrectly (false) identifies a working pump as failed (condition negative). The TPR is the ratio of all true positives to condition positives, while FPR is the ratio of false positives to condition negatives. A perfect learner would have an operating point where the true positive rate is 1 and the false positive rate is 0, but most real-world solutions are a balance between true and false positives. ROC performance of the current failure classifier is substantially better than the performance of the forecast failure classifier because it is easier to predict when pumps have already failed compared to forecasting when they will fail. For example, to achieve a true positive rate of 0.75, the current failure classifier would have a false positive rate of ∼1%, but the future failure classifier would have a false positive rate of ∼30%.

The bottom panel of Fig 2 illustrates the relationship between the cutoff threshold for failure classification and the performance of the classifier. As the threshold for determining whether a pump has failed is increased, the standard for a pump to be classified as failed becomes more difficult. This is why, as the threshold approaches 1, then true positive rate falls to zero; there are no pump days where the classifier is 100% sure that the pump is failed, so the ratio of pump days classified as failed to the days pumps are actually failed falls to zero. Similarly, as the threshold for failure classification increases, the likelihood of accurately identifying a pump as working when it is really working increases, and this is observed as a rise in the true negative rate. Positive predictive value (PPV) is the ratio of true positive classifications to all positive classifications, and negative predictive value (NPV) is the corollary. Positive predictive value generally increases with increasing threshold because the probability of of any given positive classification (failure) being true increases with the threshold, but the total number of positive classifications (PPV’s denominator) approaches zero as the threshold increases, leading to erratic behavior near a threshold of 1.

For this paper, most figures are presented over the full range of possible thresholds, but where full ranges are not presented, predictions are thresholded at 0.5 as discussed in Fig 1. A summary of learner performance for a threshold 0.5 is shown in Table 1. Table 1 also shows the performance of a solo generalized linear model (GLM) for reference against the ensemble learner. The GLM has a slightly higher true positive rate than the ensemble learner, but a much lower positive predictive value as well. This can be interpreted, roughly, as the GLM being less “nuanced” than the ensemble model with slightly more correctly-identified pump failures, but at the expense of many more incorrectly-identified pump failures.

**Table 1:** Learner performance for classifying current and forecasted failures. A solo GLM model is shown for reference against the ensemble model.

metrics	ensemble current failure	ensemble forecasted failure	GLM current failure (ref.)	GLM forecasted failure (ref.)
true positive rate (sensitivity)	85.4%	51.0%	89.5%	59.4%
true negative rate (specificity)	99.4%	99.3%	98.4%	96.5%
positive predictive value	84.8%	82.5%	69.7%	54.4%
negative predictive value	99.4%	96.7%	99.6%	97.2%

Performance values shown for a failure threshold of 0.5.

**Fig 3:**
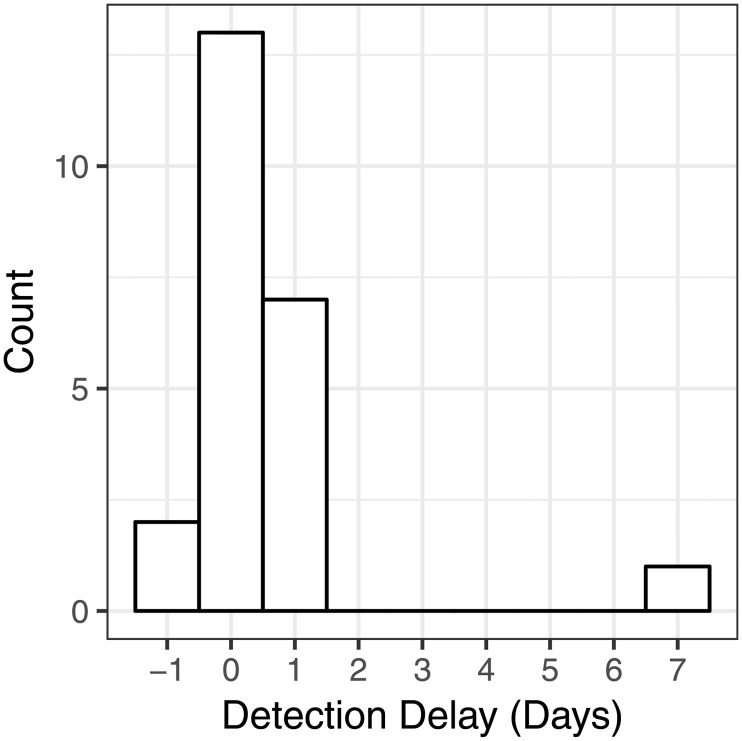
Histogram of forecasting performance. The detection delay for the 23 correctly-identified failures. Negative numbers indicate forecasted failures, 0 indicates the model detected the failure on the failure day, and positive numbers indicate detection lag. The learners in this study detected 22 out of 24 (92%) of these failure events within 1 day of failure.

Results discussed up to this point have been presented day-wise. However, most implementers consider failures in terms of strings of failure days which we call a “failure event.” So, consecutive runs of predicted failures (either current or forecast) were modeled as “prediction events.” If a predicted failure event overlapped a true failure event, that true failure event was determined to be “detected.” At a threshold of 0.5, the model was able to detect 23 of the 24 failure events in the data set. Fig 3 illustrates the model’s time delay in detecting these true failure events.

## Discussion

**Fig 4:**
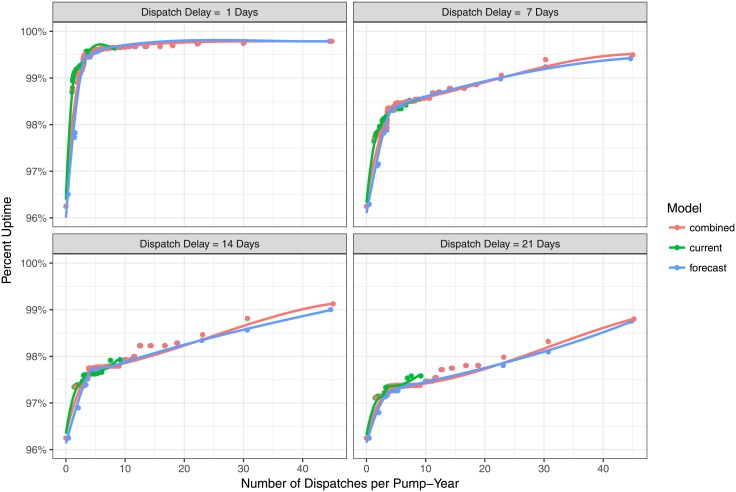
Achieving 99% uptime. The uptime of the pump fleet is plotted as a function of the number of dispatches per year for different dispatch delays. Achieving 99% uptime requires either a very short dispatch delay or many dispatches per year.

From a program viewpoint, implementers are primarily interested in increasing reliable and cost-effective water services. Fig 4 illustrates the trade-off between fleet uptime and dispatch responsiveness as a function of the number of model-initiated dispatches per pump-year. The figure is faceted by dispatch delays ranging from 1 to 21 days. There are two important insights visible in this figure. First, on a per-dispatch perspective, there is very little difference between current, forecast, and combined models. The current failure model typically performs slightly better on a per-dispatch basis (as a result of its higher positive predictive value). However, the most important difference in fleet uptime results from the implementing agency’s dispatch delay, and, to a lesser extent, the implementing agency’s capacity to perform many dispatches in a pump-year. The goal of 99% fleet uptime could be achieved with our machine learning model using just 2 dispatches per pump-year paired with a 1-day dispatch delay, or 22 dispatches per pump-year with a 7-day dispatch delay.

Table 2 estimates costs (in today dollars) of operating four different pump maintenance models in Kenya over a pump lifetime of 10 years. These maintenance models are: (1) a nominal sensor-less baseline model where pumps are repaired as-needed prompted by requests from local contacts, (2) a circuit model of sensor-less scheduled preventative maintenance where pumps are serviced on a fixed schedule, (3) an ambulance model of sensor-enabled maintenance where sensor data indicates handpump failure and initiates service events, and (4) the machine learning model described in this paper where machine learning is used to both forecast failures and/or detect failures very quickly after they happen. Although we have estimated costs for all 4 models, The Water Project currently only implements two different maintenance models in Kenya: a circuit model for pumps without sensors, and an ambulance model for pumps with sensors. Therefore, we did not directly measure the cost of implementing the nominal baseline (sensor-less as-needed maintenance) model in Kenya, but we estimate its cost by extrapolating from previous work in Rwanda [6].

**Table 2:** The cost of delivering the ML-derived preventative maintenance model compared to other models.

Category	Sub-category	Nominal baseline maintenance (no sensor, as needed)	Circuit (scheduled) maintenance (no sensor, preventative)	Ambulance maintenance (sensor-enabled, as-needed)	Machine learning maintenance (sensor-enabled, preventative)
**Capital Exp.**	Pump	$10,000	$10,000	$10,000	$10,000
	Sensor	$0	$0	$360	$360
**Annual Operational Exp.**	Sensor	$0	$0	$410	$410
	Kenya-Based Pump	$120	$230	$300	$300
	USA-Based Admin.	$730	$730	$820	$820
**NPV (5% cost of money)**	CapEx	$10,000	$10,000	$10,360	$10,360
	OpEx	$6,563	$7,413	$11,814	$11,814
	Total	$16,563	$17,413	$22,174	$22,174
**Cost of Service Delivered**	Uptime	67.5%	72.9%	96.5%	99.0%
	USD per Working Year	$2,453	$2,387	$2,298	$2,240

Note: the uptimes for the nominal baseline and circuit models were not observed in this study; uptime data for the nominal baseline and circuit models are shown for comparison from Nagel et al., 2015. Net present value calculations assume a 5% annual cost of money.

For operational expenditures, we measured that sensors cost some $110 in spare parts per sensor-year. Additionally, we estimate that running an effective machine learning-enabled program or ambulance model will require 3 extra dispatches of field staff per year: 2 false positive dispatches per year (using a 1-day dispatch delay) and 1 additional battery-swapping dispatch. We conservatively estimate that these extra dispatches will cost $100 per dispatch. We note that batteries on the sensor must actually be swapped twice per year, but we assume only one dispatch is dedicated solely to battery swapping because the other swap could be performed performed during a maintenance visit or false positive dispatch. Kenya-based pump operational expenses and USA-based administration expenses represent the staff and non-sensor-related material expenses of maintaining a pump fleet. Deploying sensors requires additional staff effort in Kenya and the USA to serve as stewards of the sensor fleet, and the associated increases in costs are shown in Table 2.

From a cost perspective, the most important comparison in Table 2 is between the sensor-less circuit (scheduled) maintenance model and the sensor-enabled machine learning model. Both of these maintenance models aim to increase fleet uptime by performing preventative maintenance. The machine learning model costs more over its lifetime to implement than the circuit model ($22,174 vs. $17,413), but the machine learning model also has significantly higher performance, boosting fleet uptime from 72.9% to 99.0%. When we adjust the cost of operating the pump to account for how much it costs to operate *per year that the pump is working*, the machine learning model is less expensive, saving 7% relative to the circuit model on the cost of water delivered.

In addition to modest savings in the direct cost of delivering water, there could be large economic and health gains realized from the high fleet uptimes enabled by machine learning. For example, prior work has shown that small improvements to pump uptime can unlock significantly higher willingness to pay: improving the downtime between failures from 27 to 2.6 days per failure (at 2 failures per year, an improvement in fleet uptime from 85% to 99%) increased willingness to pay by 5X from from 0.2 USD to 1.0 USD per day [8]. Additionally, as previously mentioned, health outcomes and access to clean water may have a highly non-linear relationship. The health benefits of drinking clean water may not be realized until clean water is highly reliable, readily available, and consumed nearly exclusively [7]. In other words, not only do sensors and machine learning make it more affordable to operate a *working* pump, sensors and machine learning also have the potential to unlock additional health benefits and financial sustainability.

The marginal cost of implementing sensors, machine learning, and preventative maintenance activity are spread over the total utility that the equipment (a handpump in this case), delivers to customers over its lifetime. For this reason, there would be an even greater per-dollar benefit from implementing a sensor and machine learning-enabled preventative maintenance program on larger commercial assets such as motorized borehole pumping stations. While the cost of sensors and algorithms would not be significantly changed, the total benefit delivered to customers per functional pump-year would be greatly increased because of the larger pumping capacity of these stations.

In conclusion, the highly non-linear relationship between pump performance and health & economic outcomes illustrates that pumps need to perform extremely well before their benefits to society can be realized. This non-linear relationship also suggests that there is more consumer surplus to be gained by improving the function of existing pumps rather than building ever more new pumps that function only marginally well. This study has demonstrated that a machine-learning-enabled preventative maintenance model has the potential to enable fleets of handpumps that function extremely well by driving total fleet uptime to >99%, thus providing a realistic path forward towards reliable and sustained clean water delivery.
